# Supplementary material for: Emergency Healthcare Providers’ Knowledge about and Attitudes toward Advance Directives: A Cross-Sectional Study between Nurses and Emergency Medical Technicians at an Emergency Department
Source: Int J Environ Res Public Health. 2021 Jan 28;18(3):1158. doi: 10.3390/ijerph18031158 (PMC7908551; doi:10.3390/ijerph18031158)
Supplement: Supplementary file 1 [file ijerph-18-01158-s001.pdf]

ADVANCE DIRECTIVE KNOWLEDGE SURVEY

**Advance directive**	**Yes**	**No**	Don't know
1. An AD can be signed by any adult.			
2. An AD is a document in which an individual indicates desired end-of-life care in advance in case he/she is no longer able to make decisions due to illness or incapacity.			
3. Family can prepare an AD on behalf of an individual.			
4. A healthcare proxy as a surrogate decision-maker can be designated on an AD.			
5. An AD can be registered in a designated agency only.			
6. A physician's or nurse's assistance is required to complete an AD.			
7. Any changes or revocation is possible when needed.			
**Life-Sustaining Treatment Plan**			
1. An LSTP is a document in which a terminal individual indicates desired end-of-life care.			
2. An LSTP is prepared by an attending physician.			
3. Terminally ill patients or patients in the dying phase can prepare an LSTP.			
4. An LSTP cannot be changed once it has been written.			
5. An LSTP can be written after discussion with the family.			
6. A DNR (do not resuscitate) order can be used instead of an LSTP.			
7. All medical care including analgesics and antibiotics is discontinued on the completion of an LSTP.			

ADVANCE DIRECTIVE ATTITUDE SURVEY

Items	4<br>Strongly Agree	3<br>Agree	2<br>Disagree	1<br>Strongly Disagree
1. I have choices about the treatment I would receive at the end of my life.				
2. I would be given choices about the treatment I would receive at the end of my life.				
3. My doctor would include my concerns in decisions about my treatment at the end of my life.				
4. If I could not make decisions, my family would be given choices about the treatment I would receive.				
5. I think my family would want me to have an advance directive.				
6. Making my end of life treatment wishes clear with an AD would keep my family from disagreeing over what to do if I were very sick and unable to decide for myself.				
7. Having an AD would make my family feel left out of caring for me.				
8. Making my end of life treatment wishes clear with an advance directive would help to prevent guilt in my family.				
9. Making my end of life treatment wishes clear with an AD would have no impact on my family.				
10. Having an AD would prevent costly medical expenses for my family.				
11. Having an AD would make sure that my family knows my treatment wishes.				
12. My family wants me to have an AD.				
13. Having an AD would make sure that I get the treatment at the end of my life that I *do* want.				
14. I trust one of my family or friends to make treatment decisions for me if I cannot make them myself.				
15. It is better to make an advance directive when you are healthy.				
16. I am not sick enough to have an advance directive.				

M. Nolan © (2003)

Cited In:

Nolan, M. T., & Bruder, M. (1997). Patients' attitudes towards advance directives and end of life treatment decisions. *Nursing Outlook, 45,* 204-208.
